# Advanced Radio Frequency Applicators for Thermal Magnetic Resonance Theranostics of Brain Tumors

**DOI:** 10.3390/cancers15082303

**Published:** 2023-04-14

**Authors:** Nandita Saha, Andre Kuehne, Jason M. Millward, Thomas Wilhelm Eigentler, Ludger Starke, Sonia Waiczies, Thoralf Niendorf

**Affiliations:** 1 Max-Delbrück-Center for Molecular Medicine in the Helmholtz Association (MDC), Berlin Ultrahigh Field Facility (B.U.F.F.), 13125 Berlin, Germany; 2 Charité—Universitätsmedizin Berlin, Experimental and Clinical Research Center (ECRC), A Joint Cooperation between the Charité Medical Faculty and the Max-Delbrück Center for Molecular Medicine in the Helmholtz Association, 13125 Berlin, Germany; 3 MRI.TOOLS GmbH, 13125 Berlin, Germany; 4 Brightmind.AI GmbH, 1010 Vienna, Austria; 5 Hasso Plattner Institute for Digital Engineering, University of Potsdam, 14482 Potsdam, Germany

**Keywords:** theranostics, hyperthermia, brain tumor, MRI, ThermalMR, RF applicator

## Abstract

**Simple Summary:**

Localized thermal therapy has been reported to have clinical benefits as a potent sensitizer of chemo and radiotherapy for various cancers, and for facilitating targeted drug delivery. Thermal magnetic resonance (ThermalMR) integrates targeted radiofrequency (RF) induced heating in the hyperthermia (HT) range, together with diagnostic MRI and in vivo non-invasive temperature mapping within a single RF applicator for thermal theranostics. The potential of dipole antenna arrays for ThermalMR and hyperthermia RF applicators is well recognized, but the additional value of loop elements remains to be investigated. Therefore, we designed circular and elliptical ThermalMR RF applicator arrays with circular 360° and horse-shoe shaped (arc = 270°) coverage of the human head, combining loop antennas and self-grounded bow-tie (SGBT) dipole antennas in a hybrid design. We investigated the performance of these designs in terms of the MRI transmission B_1_^+^ field, RF power deposition to optimize targeted RF heating, using electromagnetic field (EMF) and temperature simulations performed on a virtual patient with a clinically realistic intracranial brain tumor. ThermalMR RF applicators with the hybrid loop+SGBT dipole design showed superior MRI performance and targeted RF heating inside the tumor, while preserving healthy tissue.

**Abstract:**

Thermal Magnetic Resonance (ThermalMR) is a theranostic concept that combines diagnostic magnetic resonance imaging (MRI) with targeted thermal therapy in the hyperthermia (HT) range using a radiofrequency (RF) applicator in an integrated system. ThermalMR adds a therapeutic dimension to a diagnostic MRI device. Focused, targeted RF heating of deep-seated brain tumors, accurate non-invasive temperature monitoring and high-resolution MRI are specific requirements of ThermalMR that can be addressed with novel concepts in RF applicator design. This work examines hybrid RF applicator arrays combining loop and self-grounded bow-tie (SGBT) dipole antennas for ThermalMR of brain tumors, at magnetic field strengths of 7.0 T, 9.4 T and 10.5 T. These high-density RF arrays improve the feasible transmission channel count, and provide additional degrees of freedom for RF shimming not afforded by using dipole antennas only, for superior thermal therapy and MRI diagnostics. These improvements are especially relevant for ThermalMR theranostics of deep-seated brain tumors because of the small surface area of the head. ThermalMR RF applicators with the hybrid loop+SGBT dipole design outperformed applicators using dipole-only and loop-only designs, with superior MRI performance and targeted RF heating. Array variants with a horse-shoe configuration covering an arc (270°) around the head avoiding the eyes performed better than designs with 360° coverage, with a 1.3 °C higher temperature rise inside the tumor while sparing healthy tissue. Our EMF and temperature simulations performed on a virtual patient with a clinically realistic intracranial tumor provide a technical foundation for implementation of advanced RF applicators tailored for ThermalMR theranostics of brain tumors.

## 1. Introduction

Temperature is a physical parameter with a multitude of biological implications and a subject of great clinical interest. Fighting fire with fire, therapeutic hyperthermia (HT) (T = 40–43 °C) is an adjunct treatment of cancer therapy that can inhibit tumor growth and enhance the efficacy of other anti-cancer treatments [1,2,3,4,5,6,7,8]. Localized thermal therapy as a potent sensitizer of chemo-, radio- or immunotherapy of cancers has been reported to show clinical benefits [1,2,3,4,5,6,7,8,9,10]. Technical developments and research directions of thermal therapy have followed several trajectories, including magnetic nanoparticle hyperthermia (MNH), focused ultrasound-based hyperthermia, microwave-induced hyperthermia and radio frequency (RF)-driven hyperthermia [10,11,12,13,14,15,16,17,18,19,20,21]. With an ever-increasing clinical interest in thermal therapy, non-invasive in vivo approaches that facilitate diagnostic imaging-guided temperature therapy, along with the characterization of its subsequent effects, are imperative. An integrated platform that combines diagnosis and thermal therapy can help better define the role of temperature in biological systems and disease, and use this insight for improved thermal theranostics. There are several cutting-edge applications of thermal theranostics, including boosting the efficacy of immune-cell therapy and increasing permeability of the blood-brain barrier to allow crucial drugs to better enter the central nervous system and monitor the distribution of molecules in the interstitium [9,22,23,24,25,26]. Thermal therapy can support individualized medicine with temperature-triggered targeted drug delivery, using thermo-responsive nano-carriers for ‘smart’ theranostics [25,27,28,29]. Molecular therapy with heat-activated gene expression is a further potential application [30]. Using thermal modulation for selectively controlling neural activity patterns or genetically sensitized neurons (thermogenetics) is another compelling possibility [31].

Glioblastoma multiforme (GBM) represents 77–81% of most aggressive primary malignant tumors of the central nervous system (CNS) classified by the World Health Organization (WHO), with a median survival rate of approximately 14.6 months and no long-term cure [26,32,33,34,35,36]. Clinical studies suggest that patient survival improves when HT therapy is used as an adjunct to improve the efficacy of radio- and chemotherapy [7,8]. Adjuvant interstitial brain HT applied before and after brachytherapy was reported to significantly improve GBM patient survival (2-year survival 31% vs. 15%) with acceptable toxicity [7]. Notwithstanding the body of evidence on the effectiveness of radiotherapy + HT, thermal therapy is still not clinically widespread [1,37]. Achieving well-controlled tumor temperatures with optimal timing, sequence and dosage management constitutes major challenges of current HT applications, which limit realization of the full clinical potential of HT, especially for deep-seated tumors in the brain [1,17,38]. Current standalone RF HT approaches are constrained by limits in depth penetration, which preclude targeting deep tissue, and may be restricted to regional rather than focal heating [1,17,38]. Moreover, standalone HT approaches lack inherent in vivo non-invasive temperature mapping, real-time dose management and therapy detection capabilities [1,17,38]. 

Any in vivo HT modality strongly benefits from guidance by diagnostic imaging. Magnetic resonance (MR) is a mainstay of diagnostic imaging and an essential tool in fundamental biomedical research and clinical science. MR imaging (MRI) provides exquisite anatomical reference, facilitates functional contrast and supports non-invasive temperature mapping and therapy detection [39,40,41]. Multi-modal hybrid setups that combine MRI instruments and separate HT devices for targeted RF-induced heating using two independent RF chains have been established [42,43]. This approach requires extra hardware, is not cost effective and comes at the cost of mutual interferences between the two separate RF chains. This approach also suffers from compatibility issues and practical obstacles because each sub-device is provided by a different vendor. Thermal magnetic resonance (ThermalMR) integrates RF-induced heating in the HT range, in vivo non-invasive temperature mapping using MR thermometry (MRTh), and anatomical and functional MRI (and even physiometabolic MRI) in a single, multi-purpose RF applicator permitting supervised thermal theranostics [37,41,44,45,46,47,48,49,50,51,52,53,54]. ThermalMR adds a therapeutic dimension to a diagnostic imaging device and has great promise to address the urgent need for novel personalized patient care. 

For ThermalMR, RF antenna arrays are used to non-invasively and selectively increase the temperature of a target region [37,44,46]. ThermalMR RF applicator development and optimization is governed by the operating frequency, type and size of RF antenna, and number of antenna elements. It is also guided by the spatial arrangement of the antennas in a phased array, with the objective to ensure uniform magnetic transmission fields (B_1_^+^) for diagnostic MRI and MR thermometry, and facilitate targeted control of electric fields (E) for thermal therapy [45,46]. MRI at ultrahigh magnetic field strengths (UHF-MRI) with B₀ ≥ 7.0 T uses higher RF frequencies than conventional MRI. This permits shorter wavelengths in tissue and offers ample potential for ThermalMR-based theranostics [45]. The potential of dipole antenna arrays for ThermalMR RF applicators, as well as microwave (MW) HT applicators, is well recognized [37,44,45,46,55,56,57,58,59,60]. Trefná et al. recently documented their work on SGBT dipole antenna arrangement over the head for MW-HT [61]. Improved MRI performance of loop antennas combined with dipole antennas was shown for UHF-MRI [62,63,64,65,66,67,68,69,70]. Until now, the potential of the additional value of integrating loop elements with dipole antennas in a ThermalMR RF applicator remained to be investigated. This approach is conceptually appealing because it supports the development of high-density arrays. This could benefit ThermalMR theranostics, because an increase in the number of RF elements per unit area increases the degrees of freedom for improving transmission field (B_1_^+^) uniformity for diagnostic MRI, and enhancing targeted RF shimming and RF peak power deposition for thermal therapy. This is especially relevant for ThermalMR of GBM or other deep-seated brain tumors, since the head presents a small surface area that limits the number of RF transmission elements that can be arranged in an array to cover the head. 

Recognizing the opportunities of ThermalMR theranostics of brain tumors, we hypothesize that advanced RF applicators comprising combined loop+dipole elements will outperform the dipole-only counterparts. To test this hypothesis, we examine the suitability of hybrid loop+dipole RF applicator concepts for ThermalMR at 300 MHz (7.0 T), 400 MHz (9.4 T) and 450 MHz (10.5 T). For this purpose, we use a compact self-grounded bow-tie (SGBT) dipole antenna integrated with a loop element to form a loop+dipole building block. Eight hybrid loop+SGBT dipole building blocks are arranged in arrays to facilitate MRI and constructive E-field focusing in the target region of the brain. ThermalMR RF applicators with different spatial arrangements of the loop+SGBT building blocks are examined including circular and elliptical array configurations using 360° or horse-shoe-like 270° coverage of the brain. We hypothesize that the horse-shoe configurations are superior to the 360°-coverage configurations, and would improve patient safety and comfort by eliminating RF building blocks close to the nose, chin and eyes. To assess the performance of the ThermalMR RF applicators in a realistic setup, we performed electromagnetic field (EMF) and temperature simulations in a human voxel model, modified by including a small brain tumor replicating a clinical scenario. 

## 2. Materials and Methods

**Figure 1:**
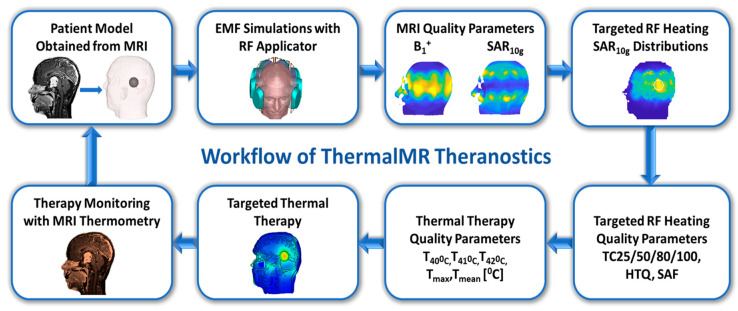
Scheme of thermal MR treatment planning (TMTP). B_1_^+^ = transmission field used for MRI; TC = tumor coverage; T = temperature; SAR = specific absorption rate; SAF = SAR amplification factor; HTQ = hotspots-to-target quotient.

Numerical EMF simulations using optimization algorithms in virtual patient models provide a springboard and technical foundation for the development of advanced ThermalMR RF applicator concepts customized for simultaneous MRI and RF hyperthermia for ThermalMR theranostics [71]. Realistic virtual patient models incorporated into the EMF simulations provide essential information about the performance of ThermalMR RF applicators with respect to the MRI characteristics and RF power deposition, i.e., specific absorption rate (SAR) and temperature distributions in the target regions of the brain. The workflow of ThermalMR theranostics including diagnostic imaging, treatment planning (TMTP), thermal therapy and therapy detection is presented in Figure 1. 

### 2.1. Electromagnetic Field Simulations and Patient Model

**Figure 2:**
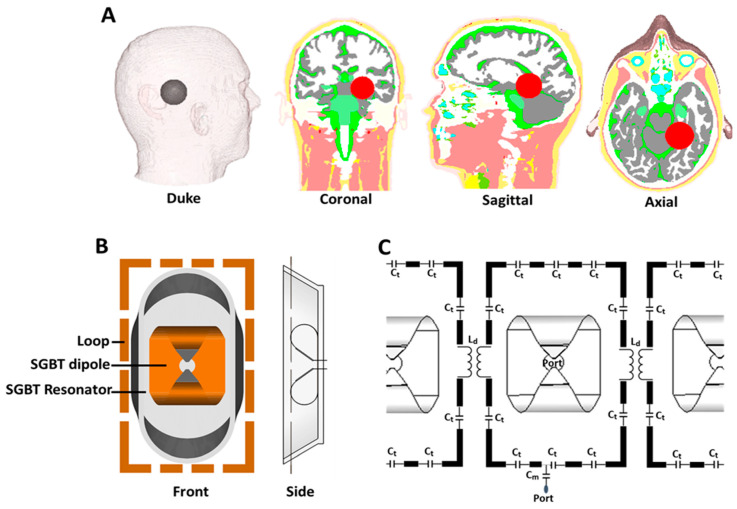
(**A**) Human voxel model ‘Duke’ from the virtual family IT’IS modified with a small brain tumor (red) in the right parietal region with coronal, sagittal, axial views [72]. (**B**) Compact SGBT dipole and loop building block. The wideband self-grounded bow-tie (SGBT) dipole antenna has a range of 250–600 MHz operating frequencies. (**C**) Schematic of loop+SGBT building blocks configured in an array. Loops were tuned to be multi-resonant to work with wideband SGBT dipoles at frequencies of 250, 300, 350, 400, 450 MHz. The loops are decoupled from each other with a transformer decoupling L_d_ [80]. C_m_ and C_t_ are matching and tuning capacitors of loops.

The human voxel model ‘Duke’ from the virtual family (IT’IS Foundation Zürich, Switzerland) was used for EMF simulations and TMTP [72]. TMTP is based on EMF results simulated with CST Microwave Studio Suite 2020 (Dassault Systèmes Darmstadt, Germany) using the time-domain solver based on the finite integration technique (FIT). To reduce the computational effort of the EMF simulations, the human voxel model (maximum resolution of 2.0 × 2.0 × 2.0 mm^3^) was truncated at the level of the neck (Figure 2A).

To mimic a realistic clinical scenario, the Duke model was modified with an intracranial sphere (radius = 2 cm) that represents a small tumor in the right parietal region of the brain, with a target volume (TV) of 33.5 mL [37]. The dielectric and thermal properties of the tumor model were assigned based on the literature reports on human glioma where tumor perfusion is decreased by a factor of 0.7 [37,73,74]. The dielectric and thermal tissue properties of the remaining healthy tissues were assigned according to the database provided by the IT’IS Foundation [75]. To accelerate the simulation speed of broadband simulation by not modeling materials as dispersive, tissue dielectric properties were taken from the IT’IS database at 350 MHz as tissue dielectric properties do not vary strongly in the frequency range of interest. The dielectric and thermal properties of the tumor and healthy tissues used for the human voxel model Duke are summarized in Supplementary Table S1.

### 2.2. Integration of Loop and Dipole Antenna in a Hybrid RF Building Block

Loop antennas are single-frequency resonant, show asymmetric B_1_^+^ field distribution and provide good surface E-fields [62,63,64,65,66]. Dipole antenna arrays tailored for MRI offer uniform B_1_^+^ fields, can operate over a broadband frequency range and provide a central E-field distribution compared to loop arrays [62,63,64,65,66,67]. The current distribution of a dipole antenna is symmetric along its long axis, whereas a loop antenna demonstrates an antisymmetric current distribution [64,65,66]. Given the distinct current distribution patterns of loops and dipoles, mutual element decoupling can be achieved with loop+dipole combinations [62,63,64,65,66]. This provides an opportunity to develop advanced ThermalMR RF applicators.

Recognizing the opportunity, we implemented combined loop+dipole RF building blocks in EMF simulations. For this purpose, we used an ultra-wideband (range 250–650 MHz) self-grounded bow-tie (SGBT) dipole antenna (size: 42.3 × 46.3 × 2.5 mm^3^) adapted for ThermalMR (Figure 2B,C) [76]. The SGBT antennas were fed at the center. Each SGBT antenna uses a water bolus placed between the radiating element and the surface of the object under investigation to enhance the efficiency and directivity for targeted RF heating [77,78,79]. The water bolus was modeled as the shape of the outer surface of the SGBT antenna resonator and positioned between the SGBT and Duke’s head. To form a combined loop+SGBT dipole building block, the SGBT antenna was integrated with a rectangular loop antenna, illustrated in Figure 2B,C. Rectangular loop elements were modelled using 5 mm wide copper strips (size: 75 × 125 × 1 mm^3^). The loops were fed with a capacitive matching (C_m_) network and decoupled from each other with a transformer decoupling L_d_ (Figure 2B,C) [80]. Eight fixed capacitors were distributed over the length of the loop to serve as tuning capacitors (C_t_). The loops and SGBT dipoles were individually tuned and matched to support RF-induced target heating at multiple discrete frequencies: 250, 300, 350, 400, 450 MHz. The loop+SGBT building block will be further referred to as LD.

### 2.3. ThermalMR RF Applicators

**Figure 3:**
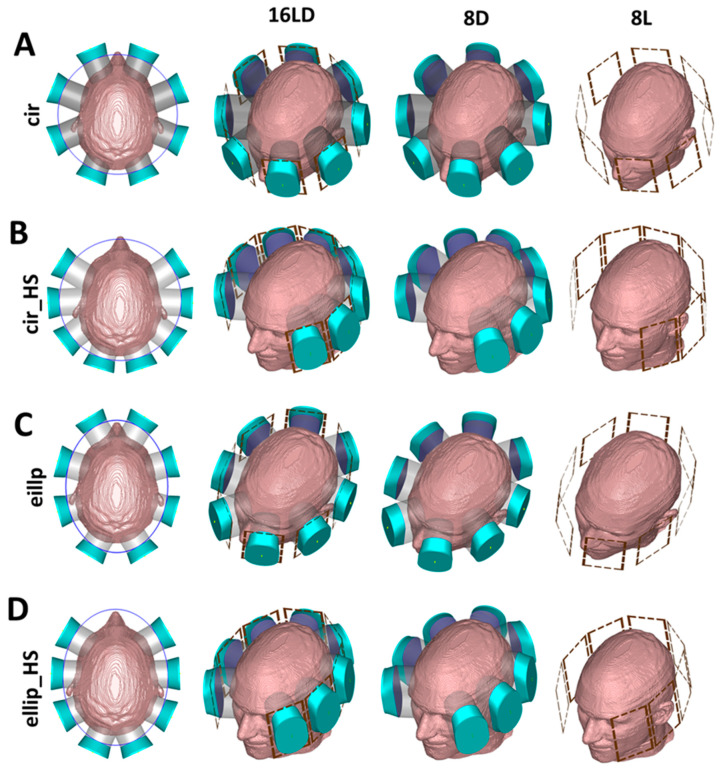
Overview of ThermalMR RF applicator configurations with 16LD, 8D and 8L variants around the head of Duke. Circular and elliptical array configurations were designed using 360° coverage, and the horse-shoe shape using arc = 270° coverage of the head. Arrangements of the loop+SGBT dipole building blocks are illustrated on the left column: (**A**) circular array (cir), (**B**) circular horse-shoe array (cir_HS), (**C**) elliptical array (ellip), (**D**) elliptical horse-shoe array (ellip_HS).

ThermalMR RF applicators were designed as an array of eight LD building blocks (hereafter referred to as ‘16LD’: 8 loops and 8 dipoles) positioned around the head of the human voxel model Duke (Figure 3A–D). The 16LD array is fed by 16 separate RF transmission channels. Two 16LD arrays with 360° coverage around the head were implemented: circular (cir, radius = 130 mm) and elliptical (ellip, radius_major axis_ = 130 mm, radius_minor axis_ = 100 mm) configurations (Figure 3A–C). Alternatively, two horse-shoe shaped circular (cir_HS) and elliptical (ellip_HS) arrays were designed, in an arc of 270° to ensure ample brain coverage while sparing the high conductivity regions of the eyes from RF exposure during targeted heating (Figure 3B–D). 

All ThermalMR RF applicators support MRI at 300 MHz (B₀ = 7.0 T), 400 MHz (B₀ = 9.4 T) and 450 MHz (B₀ = 10.5 T) and RF-induced targeted heating using multiple discrete frequencies of 250, 300, 350, 400, 450 MHz. For best MRI performance, the magnetic transmission field B_1_^+^ should be perpendicular to the static magnetic field (B₀). Therefore, the LD building blocks were arranged with their long axis parallel to the head-feet (cranial-caudal) direction. Each LD was positioned around the head of the human voxel model so that the brain is centered in the x–y-plane (left–right, anterior–posterior) of the array and the TV is centered in the z-direction (superior–inferior) of the array. For comparison, ThermalMR RF applicator arrays with identical geometry and configuration to that of the 16LD, but comprising only eight SGBT dipole antennas (referred to as 8D) or only eight loop elements (referred to as 8L) were also implemented and evaluated in the EMF simulations (Figure 3A–D).

### 2.4. Transmission Field and RF Power Deposition for MRI

To assess the MRI capability of the designed ThermalMR RF applicators, we examined the B_1_^+^ distribution and RF power deposition (SAR_10g_). Post-processing of B_1_^+^ and RF power deposition was performed in MATLAB 2020 (The Mathworks, Natick, CA, USA). The B_1_^+^ distribution was calculated for the circularly polarized (CP) mode at magnetic field strengths of B₀ = 7.0 T, 9.4 T and 10.5 T, for 1 W forward power [81]. In the CP mode, each channel of the RF applicator is driven by a phase corresponding to its angular position relative to the center of the head in the transverse plane [82]. The transmission field (B_1_^+^) efficiency (given in µT/√kW) and SAR_10g_ were compared for all ThermalMR RF applicators within a spherical region of interest (ROI, radius = 2 cm) centered in the human voxel model Duke’s head, for all three magnetic field strengths. RF power deposition was measured based on the specific absorption rate normalized to 1 W input power and averaged over 10 g of tissue (SAR_10g_). Safety guidelines governed by the IEC standard 60601-2-33 restrict the maximum SAR_10g_ in 1st level operating mode to 3.2 W/kg (volume coil) and 20 W/kg (local transmit coil) for the whole head [83].

### 2.5. Targeted RF Heating for Thermal Therapy

**Figure 4:**
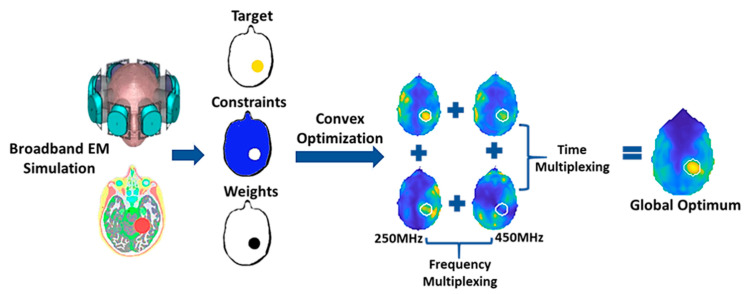
Multiplexed vector field shaping (MVFS) optimization [84]. The optimization process is a convex formulation of the time-frequency-multiplexed constrained RF heating problem, and provides an iterative algorithm to efficiently find its globally optimum solution. The MVFS algorithm provides globally optimal excitation vectors by defining the appropriate phase and amplitude settings for each RF channel of the ThermalMR RF applicator, and automatically selects the appropriate intervention frequencies and time-interleaved excitations to match the constructive interference pattern to best deliver RF power at the desired target location and shape. The resulting SAR distribution of the incident E-field interference is tailored to focus heating of the target volume while minimizing local peak RF exposure to healthy tissue below a safe threshold. If M individual excitation modes belong to the same excitation frequency, then time multiplexing is done with each solution vector scaled by √M, and the excitations were played out sequentially [84]. Excitations at different frequencies can be played out concurrently as their electromagnetic fields do not interact coherently [84]. The final resultant target shape is created by superimposing these individual patterns for each frequency.

Given the great complexity of thermoregulation in vivo, assessment of SAR_10g_ as a common metric of local RF power deposition is essential before proceeding with any thermal intervention [71,73,84]. We used a time and frequency multiplexed vector field shaping (MVFS) algorithm to optimize local RF power deposition for targeted RF heating (Figure 4) [84]. The optimization algorithm provided globally optimal excitation vectors, by defining the phase and amplitude settings for each RF channel of the ThermalMR RF applicator. The MVFS algorithm automatically selects the appropriate intervention frequencies and time-interleaved excitations to best deliver RF power at the desired target shape and location. The resulting SAR distribution of the incident electric fields interference is tailored to focus heating of the TV while minimizing local peak RF exposure to healthy and remote tissue below a defined threshold (constraint SAR). Excitation vectors for each frequency are termed as the excitation mode (M). If M individual modes belong to the same excitation frequency, then time multiplexing is done with each solution vector scaled by √M, and the excitations were played out sequentially. Excitations at different frequencies can be played out concurrently as their electromagnetic fields do not interact coherently. The final resultant target shape is created by superimposing the individual patterns for each frequency.

Multiple discrete frequencies of 250, 300, 350, 400, 450 MHz were used to optimize RF-targeted heating using time and frequency multiplexing. The desired target SAR was set to 100 W/kg to maximize RF power deposition inside the tumor TV. SAR was constrained for regions outside the TV, i.e., the safe limit for healthy tissue: SAR_10g,max(healthy tissue)_ = SAR_constraint_ = 40 W/kg.

(1) $$Cₚ\rho \frac{\partial T}{\partial t}=\nabla \cdot \left(k\nabla T\right)-C_{b}W_{b}\left(T-T_{a}\right)+PLD$$

The SAR_constraint_ limit was decided based on simplifying the temperature rise models using the Pennes’ bioheat transfer equation [85]. In Equation (1), C_p_ is the specific heat capacity (J/kg/°C); ρ is the tissue density (kg/m^3^); k is the thermal conductivity (W/m/*°*C); C_b_ is the specific heat capacity of blood (J/kg/*°*C); W_b_ is the volumetric perfusion rate (kg/m^3^/s); T_a_ is the local arterial or body core temperature; PLD is power loss density (W/m^3^) deposited by the heating system.

In steady-state formulation of the Pennes’ bioheat Equation (1), the time derivative on the left-hand side can be set to zero. The first transient term on the right-hand side can be neglected, as the characteristic time of temperature adaptation is short compared to the duration of the thermal therapy. Absent any time or temperature-dependent properties, the remaining terms (T − T_a_) govern the temperature (T) increase. With a basal blood perfusion (W_b_) of 50 mL/100 g/min, an average SAR of 50 W/kg results in a temperature change (T − T_a_) of ~∆T ± 1.5 *°*C [37] without considering the response of a thermoregulatory perfusion (C_b_) increase. Therefore, we set the SAR_constraint_ at 40 W/kg to be in accordance with previous studies that used a SAR of approx. 20–40 W/kg, and reported a temperature increase to ~42 °C in the target region [86,87].

### 2.6. Temperature Simulations

Temperature distribution for 16LD ThermalMR RF applicators were calculated using the thermal transient solver of CST Microwave Studio Suite 2020, (Dassault Systèmes, Darmstadt, Germany). At first, the steady state temperature without any RF heating, i.e., basal temperature level or initial body temperature, was calculated using the thermal steady state solver at an ambient temperature T = 25 °C [87,88,89,90]. The initial temperature of the water boluses was fixed at T = 25 °C. Subsequently, the transient heating phase was evaluated for an intervention period of 30 min using the thermal transient solver considering isothermal boundaries with the goal to evaluate the relation between total achieved SAR_10g_ and overall temperature rise in the TV from the basal body temperature level [87,88,89,90]. To simulate the effect of RF heating, the temperature was calculated from the power loss density W/m^3^ (local SAR_10g_) based on the phase and amplitude setting provided by the MVFS algorithm of the targeted RF heating optimization. The total power applied was the sum of the individual excitation mode power obtained from the targeted RF heating optimization for all 16LD ThermalMR RF applicators. The total power used in the temperature simulation is summarized in Table 1.

### 2.7. Thermal Therapy Quality Assessment

We evaluated the performance of the ThermalMR RF applicators using clinically relevant quality indicators provided by the European Society for Hyperthermic Oncology (ESHO) according to the criteria of characterization of the SAR_10g_, index temperature distribution inside TV and hotspots in healthy tissue [73,91,92,93,94,95,96]. These quality indicators allow the investigation of localized and averaged RF power deposition in the target volume, as well as in remote healthy tissue. 

SAR_TV_: SAR_TV_ (mean SAR_10g_ within the TV) has been shown to be predictive of therapeutic outcomes of thermal treatment [92,93]. 

SAR_max(TV)_: SAR_max(TV)_ reflects the maximum absolute RF power deposition, and is directly related to the quality of the heating intervention [92,93]. 

Tumor coverage (TCx): Tumor coverage (TCx), or iso-SAR target coverage, is defined by the following Equation (2):

(2) $$TCx=\frac{({V_{TV})}_{x\%SARmax}}{V_{TV}}$$

representing the fraction (percentage) of the target volume (V_TV_) enclosed by x% peak SAR_max_ isolines, i.e., TC25, TC50, TC80 and TC100 indicate the fraction of the tumor volume enclosed within the 25, 50, 80 and 100% isolines of peak SAR_max_, respectively [73,91,93,94]. Tumor coverage reflects the homogeneity of RF power deposition inside a tumor, and TC25 > 75% is typically considered for clinical treatment [73,91,93,94]. 

Hotspots-to-target quotient (HTQ): The hotspots-to-target quotient (HTQ) is the ratio of average SAR_10g_ in the first percentile (P1) of healthy voxels exposed to the highest SAR_10g_ versus the average SAR_10g_ in the TV:

(3) $$HTQ=\frac{P1,mean\left({SAR}_{10g}\left(Healthy\right)\right)}{mean\left({SAR}_{10g}\left(TV\right)\right)}$$

The HTQ reflects hotspot sizes, focusing on local maxima creation relative to the TV [37,73,91,93,94]. Values of HTQ ≤ 1 are typically considered acceptable for clinical treatment [73]. 

SAR amplification factor (SAF): The SAR amplification factor is the ratio of average local SAR_10g_ in the TV to the average local SAR_10g_ of healthy tissue [37,92,93,94]:

(4) $$SAF=\frac{mean\left({SAR}_{10g}\left(TV\right)\right)}{mean\left({SAR}_{10g}\left(Healthy\right)\right)}$$

A higher SAF indicates less unwanted exposure of healthy tissue to RF power deposition, but does not provide information about specific local maxima or absolute RF power deposition levels [37]. In clinical routines, a SAF > 1 is considered desirable [92,93].

T_mean_, T_40°C_, T_41°C_, T_42°C_: T_mean_ is the mean temperature in the tumor TV, and the cumulative minutes (cumin) for T_mean_ > T_Basal_ (the basal body temperature) is an indicator of the heating efficiency [87,93,94]. Index temperature coverage (T_X°C_) is evaluated as the percent of the tumor volume covered with 40 °C (T_40°C_), 41 °C (T_41°C_), 42 *°*C (T_42°C_) [87,91,93]. 

### 2.8. Statistical Analysis

Statistical analysis was done for the MRI transmission field B_1_^+^ and optimized targeted RF heating results inside the tumor TV for all ThermalMR RF applicators. Data were evaluated for Gaussian distribution using the Shapiro-Wilk test. Differences in the metrics among the ThermalMR RF applicators were analyzed using the non-parametric Kruskal-Wallis ANOVA, followed by the Dunn’s post-hoc test with correction for multiple comparisons. Data were analyzed using the statistical environment R v.3.6.3; *p*-values < 0.05 were considered significant.

## 3. Results

### 3.1. Transmission Field and RF Power Deposition for MRI

**Figure 5:**
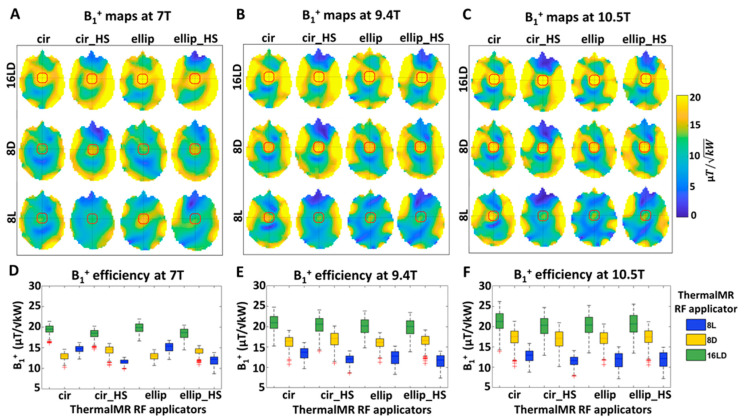
B_1_^+^ distribution maps (central axial slice of the head of the human voxel model Duke) of 16LD, 8D and 8L ThermalMR RF applicators cir, cir_HS, ellip, ellip_HS at magnetic field strength of (**A**) 7.0 T, (**B**) 9.4 T, (**C**) 10.5 T in circular polarization mode for a spherical region of interest (ROI) (radius = 2 cm, red circle) centered in the head of the human voxel model Duke. All four 16LD ThermalMR RF applicators show enhanced B_1_^+^ distribution inside the ROI compared with the corresponding 8D and 8L ThermalMR RF applicators. Comparison of the B_1_^+^ efficiency in the ROI for the 16LD, 8D and 8L ThermalMR RF applicators variants cir, cir_HS, ellip, ellip_HS at field strengths (**D**) 7.0 T, (**E**) 9.4 T, (**F**) 10.5 T shows increasing mean and maximum B_1_^+^ with increasing magnetic field strength, with reduced B_1_^+^ homogeneity. The 16LD variants had significantly higher B_1_^+^ compared to the 8D and 8L variants (see Table 2).

The B_1_^+^ transmission field distribution is shown for a central-axial slice through the head of the human voxel model Duke for all ThermalMR RF applicators at B₀ = 7.0 T (Figure 5A), 9.4 T (Figure 5B) and 10.5 T (Figure 5C). For all arrays, the 16LD ThermalMR RF applicators had higher B_1_^+^ efficiency than the 8D and 8L arrays, indicating superior MRI performance. 

At 7.0 T the 16LD_cir_, 16LD_ellip_, 16LD_cir_HS_ and 16LD_ellip_HS_ variants showed an improvement in mean B_1_^+^ efficiency of 51, 28, 53 and 30% over the 8D variants, and 58, 58, 31 and 54% over the 8L variants. The 16LD_cir_ and 16LD_ellip_ variants yielded improved B_1_^+^ homogeneity in terms of maximum and mean B_1_^+^ versus the 16LD_cir_HS_ and 16LD_ellip_HS_ counterparts. The 16LD_ellip_ variant provided the best overall maximum B_1_^+^ efficiency. At 9.4 T, the 16LD_cir_, 16LD_ellip_, 16LD_cir_HS_ and 16LD_ellip_HS_ variants showed an improvement in mean B_1_^+^ efficiency of 29, 20, 25 and 20% over the 8D variants, and 46, 71, 61, and 72% increase over 8L variants. At 10.5 T the 16LD_cir_, 16LD_ellip_, 16LD_cir_HS_ and 16LD_ellip_HS_ variants yielded an improvement in mean B_1_^+^ efficiency of 22%, 19%, 19% and 19% over the 8D variants, and 35%, 74%, 73% and 74% over the 8L variants. Mean, maximum and minimum values for each ThermalMR RF applicator are listed in Table 2.

Analysis of the B_1_^+^ efficiency achieved inside the ROI shows that the 16LD had statistically significant higher B_1_^+^ efficiency compared to the 8D and 8L variants, for all configurations at B₀ = 7.0 T (Figure 5D), 9.4 T (Figure 5E) and 10.5 T (Figure 5F) (*p*-values are listed in Table 2).

**Figure 6:**
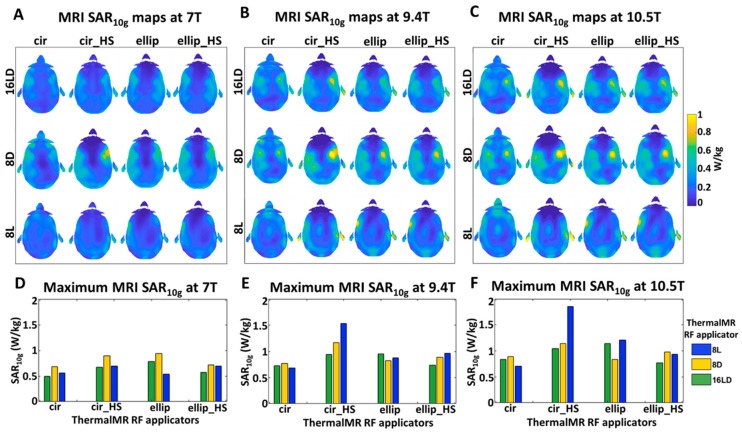
Maximum local SAR_10g_ does not exceed the IEC limits for all ThermalMR RF applicators for MR imaging. SAR_10g_ maps of the head of the human voxel model Duke (central axial slice) of ThermalMR RF applicators 16LD, 8D and 8L with cir, cir_HS, ellip, ellip_HS variants at magnetic field strength (**A**) 7.0 T, (**B**) 9.4 T, (**C**) 10.5 T. Quantification of maximum local SAR_10g_ at (**D**) 7.0 T, (**E**) 9.4 T, (**F**) 10.5 T.

Aside from the intentional power deposition desired for targeted RF heating, the suitability of the ThermalMR applicators for MRI requires control of undesired power deposition. The SAR_10g_ deposition maps obtained for a central-axial slice of Duke’s head for all ThermalMR RF applicators at B₀ = 7.0 T (Figure 6A,D), 9.4 T (Figure 6B,E) and 10.5 T (Figure 6C,F) show that in every case the SAR_10g_ is well within the safe limits defined by the IEC standard. The 8L_cir_HS_ variant showed the highest maximum local SAR_10g_ = 1.8 W/kg. 

To summarize, the EMF simulations show that all 16LD variants provide MRI transmission fields and a safe limit of RF power deposition (SAR_10g_), which are very well suited for MRI at 7.0 T 9.4 T and 10.5 T.

### 3.2. Targeted RF Heating for Thermal Therapy

**Figure 7:**
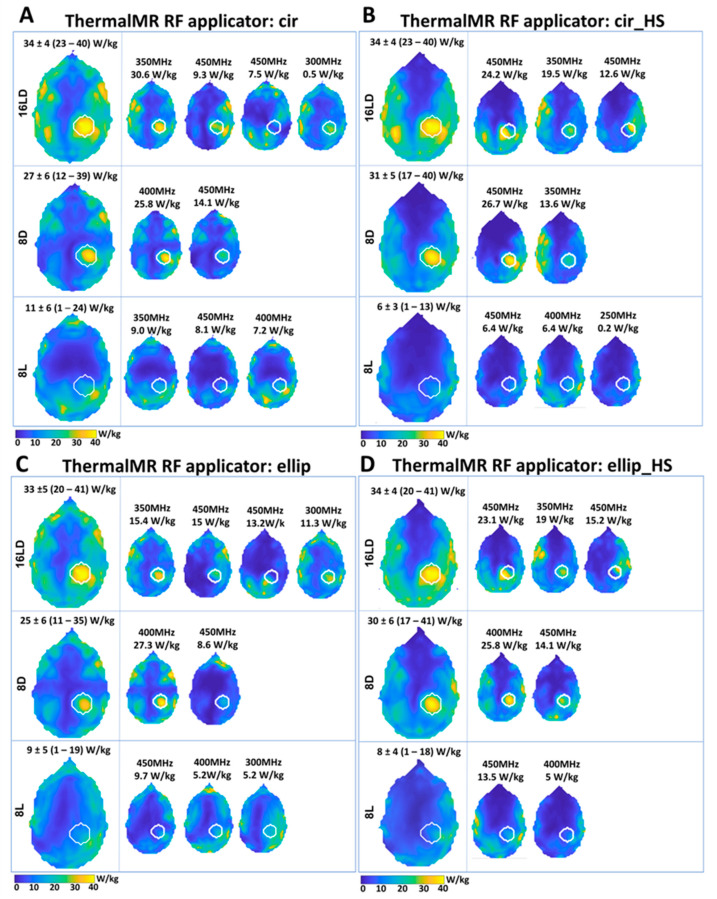
Optimization results obtained for targeted RF heating using the MVFS algorithm. Results are shown for frequencies of 250, 300, 350, 400, 450 MHz on the head of the human voxel model Duke (axial slice) for each ThermalMR RF applicator (16LD, 8D, 8L) with the four variants: (**A**) cir, (**B**) cir_HS, (**C**) ellip, (**D**) ellip_HS. Each panel shows a central axial slice through the tumor center; the targeted tumor region (TV) is depicted with a white line. The larger plots on the left of each panel shows the total achieved SAR_10g_ in the tumor TV (the mean ± SD and range (min–max value) is indicated above each plot. The resultant target pattern was created from the individual contributing time and frequency-multiplexed modes, shown in the smaller plots on the right sides of each panel (scaled to their individual maxima), with the respective peak contribution inside the tumor TV indicated above.

Results of targeted RF heating optimization with MVFS algorithm of the spherical tumor of Duke’s head with the individual excitation modes (small maps) and their combined results (large maps) are shown in Figure 7. For 16LD_cir_ and 16LD_ellip_, the MVFS algorithm determined that four excitation modes (M) at three distinct frequencies (300, 350, 450 MHz) was the optimum solution to create the resultant SAR pattern (Figure 7A,C). For 16LD_cir_HS_ and 16LD_ellip_HS_ , three modes at two distinct frequencies (350, 450 MHz) were found as the optimum solution (Figure 7B,D). In cases where two modes were found at the same frequency (i.e., 450 MHz), these were added to the resultant total SAR_10g_ by frequency multiplexing. For the 8D RF applicators, the algorithm found two excitation modes at 400 and 450 MHz (8D_cir_, 8D_ellip_ and 8D_ellip_HS_, Figure 7A,B,D) and 350 and 450 MHz (8D_cir_HS_, Figure 7C). For the 8L variants 8L_cir_, 8L_cir_HS_ and 8L_ellip_, three modes at three distinct frequencies were found (Figure 7A–C), and for 8L_ellip_HS_ , two modes at 400 MHz, 450 MHz were found (Figure 7D).

The targeted RF heating optimization local SAR_10g_ maps show that the 16LD and 8D variants are clearly able to deposit RF power inside the tumor TV (Figure 7A–D). Nevertheless, the 8D variants show lower RF power deposition inside the TV and less tumor coverage than the 16LD variants. The 8L variants show even worse performance, with substantially less power deposition, which is mainly concentrated at the periphery of the tumor and head regions due to the limited penetration depth of loop antennas.

### 3.3. Temperature Simulations

**Figure 8:**
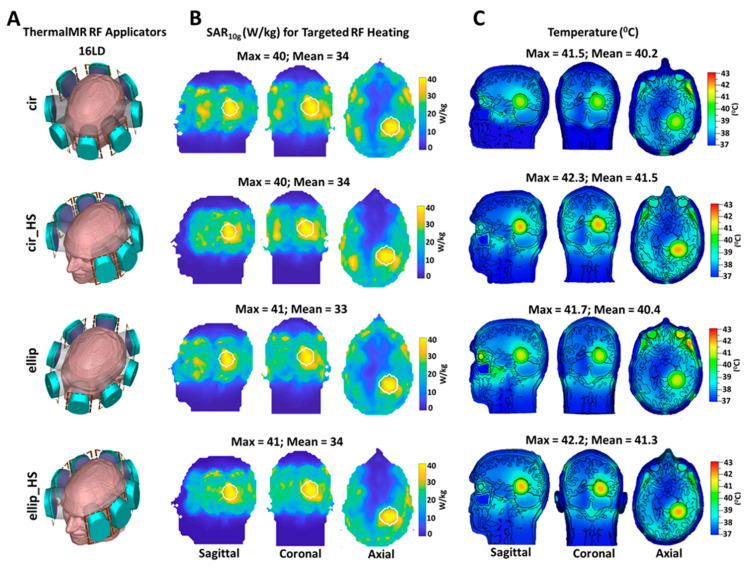
SAR_10g_ of targeted RF heating optimization and temperature distribution maps obtained for the 16LD ThermalMR RF applicators. (**A**) Diagram showing the placement of the 16LD arrays around the head. (**B**) Maps of total local SAR_10g_ achieved within the tumor target volume (white line) from targeted RF heating optimization; sagittal, coronal, axial views. The maximum (max) and mean SAR_10g_ in the target volume is indicated above the maps. (**C**) Temperature distribution maps based on total achieved SAR_10g_ results obtained from targeted RF heating optimization. T = 37.4 °C was used as a baseline body temperature. Maximum (max) and mean temperature (°C) is indicated above the maps.

The four 16LD ThermalMR RF applicators are shown in Figure 8A. A detailed view of the SAR_10g_ distribution maps obtained for the 16LD ThermalMR RF applicators is shown for sagittal, coronal and axial orientations (Figure 8B). The steady-state temperature distribution maps derived from the temperature simulations for the four 16LD ThermalMR RF applicators are shown in Figure 8C. The temperature maps show that both horse-shoe variants (16LD_cir_HS_ and 16LD_ellip_HS_) achieved the most uniform focal tumor TV heating, with maximum temperatures (T_max_) of 42.3 °C and 42.2 °C, respectively. The 16LD_cir_ and 16LD_ellip_ variants achieved T_max_ = 41.5 °C and T_max_ = 41.7 °C, respectively, in the tumor TV. However, the 16LD_cir_ and 16LD_ellip_ RF applicators also produced undesired temperature hotspots (T_max_ = 42.4 °C and T_max_ = 43 °C) in the remote facial muscles located close to the right eye. The best heating performance was provided by the 16LD_ellip_HS_ RF applicator, which showed the least facial muscle heating (T_max_ < 40 °C) and the best tumor TV heating (T_max_ = 42.2 °C and T_mean_ = 41.3 °C). 

**Figure 9:**
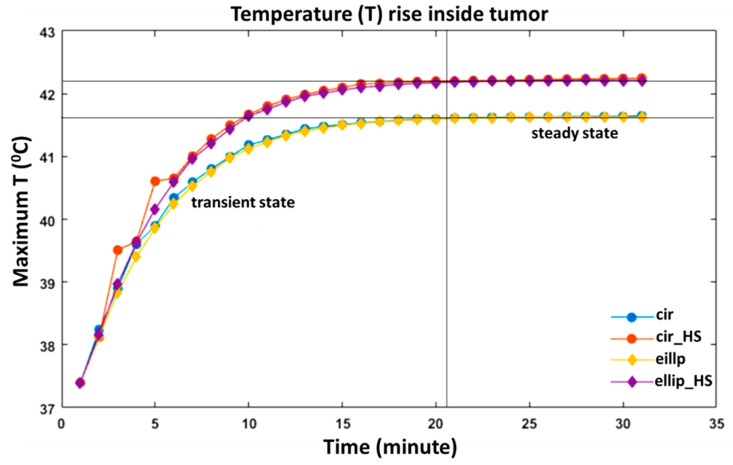
Maximum temperature (T_max_) obtained in tumor TV with respect to RF exposure time with deposited RF power (SAR_10g_) calculated from targeted RF heating optimization. For all 16LD ThermalMR RF applicators, T_max_ of the tumor TV reached a steady state after ~20.5 min. The 16LD_cir_ and 16LD_ellip_ yielded a steady state T_max_~41.7 °C and the horse-shoe 16LD_cir_HS_ and 16LD_ellip_HS_ achieved a steady state T_max_~42.3 °C.

The transient heating paradigm, which illustrates the kinetics of the maximum tumor TV temperature as a function of the RF power exposure time is illustrated in Figure 9. For all 16LD RF applicators, T_max_ of the tumor TV reached an equilibrium at t = 20.5 min. The 16LD_cir_HS_ and 16LD_ellip_HS_ arrays showed a temperature rise in the tumor TV from 37.4 °C basal body temperature to a maximum ~42.2 °C with SAR_10g_ of ~40 W/kg. For the same heating paradigm, a temperature increase from basal body temperature (37.4 °C) to T*_max_* of ~41.5 °C in the tumor TV was observed for the 16LD_cir_ and 16LD_ellip_ arrays. This approach suits thermal dose management using the cumulative equivalent minutes (CEM) 43 °C method [17,65,89].

### 3.4. Thermal Therapy Quality Assessment

**Figure 10:**
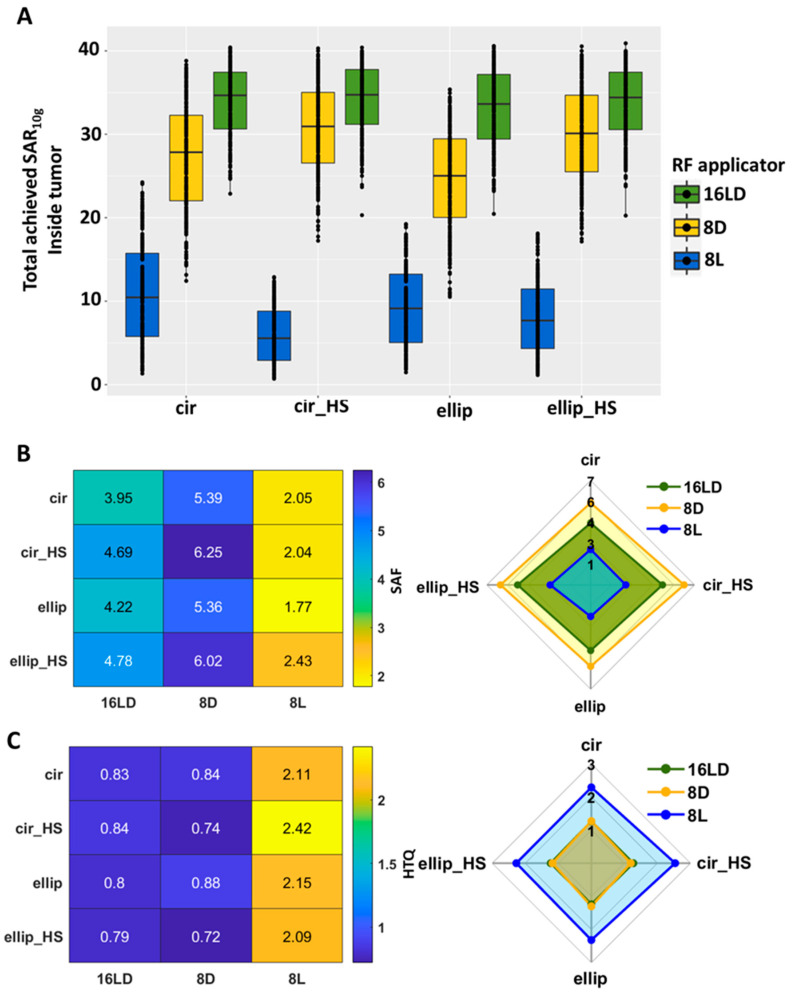
Quantification of SAR_10g_ of targeted RF heating optimization, SAR amplification factor (SAF), and hotspot-to-target quotient (HTQ). (**A**) The 16LD ThermalMR RF applicators showed significantly greater total SAR_10g_ inside the tumor TV compared with the 8D and 8L RF applicators. The 16LD_cir_, 16LD_cir_HS_, 16LD_ellip_, 16LD_ellip_HS_ ThermalMR RF applicators show a 24%, 12%, 14% and 34% increase of mean SAR_10g_ over 8D variants. Statistical analysis shows for the cir_HS ThermalMR RF applicator variant that SAR_10g_ was 34.7 ± 6.57 (median ± IQR) compared with 30.9 ± 8.47 for 8D, and 5.57 ± 5.89 for 8L. This result was consistent for the cir, ellip and ellip_HS (Table 3). (**B**) SAR amplification factor (SAF) presents the ratio of mean SAR_10g_ deposited in the target volume (TV) versus the surrounding healthy tissue. A heatmap shows the SAF across all ThermalMR RF applicators (left). Color ranges from blue (cooler) to yellow (warmer); blue indicates better preservation of remote healthy tissue from SAR exposure. The 16LD and 8D ThermalMR RF applicators achieved higher SAF than the 8L ThermalMR RF applicators, indicating better RF power deposition (SAR_10g_) inside the TV and better sparing of remote healthy tissue (right). All 8L ThermalMR RF applicators showed SAF~2. Comparing among variants of the 16LD, 8D, 8L ThermalMR RF applicators show that both horse-shoe ThermalMR RF applicators achieved higher SAF than the 360° RF applicators (right). (**C**) Heatmap of hotspot-to-target quotient (HTQ) of all ThermalMR RF applicators showing that the ellip_HS variant had better HTQ (left). Color ranges from blue (cooler) to yellow (warmer); blue indicates better performance with regards to hotspots. Comparison among all ThermalMR RF applicators shows that both the 16LD and 8D ThermalMR RF applicator have lower values (~0.88) compared to the 8L (left).

SAR_TV_ evaluation showed that the 16LD ThermalMR RF applicators had significantly greater SAR_TV_ compared with the 8D and 8L variants (Figure 10A). The SAR_TV_ obtained for the 16LD_cir_, 16LD_cir_HS_, 16LD_ellip_, 16LD_ellip_HS_ ThermalMR RF applicators was ~34 W/kg, representing an improvement of 24, 12, 14 and 34% versus the 8D counterparts. The 8L_cir_, 8L_cir_HS_, 8L_ellip_, 8L_ellip_HS_ RF applicators achieved substantially less SAR_TV_ compared to the 16LD and 8D variants. The values of the mean, maximum, minimum SAR_TV_, and *p*-values for the statistical comparison are listed in Table 3. 

SAR_max(TV)_ analysis demonstrated that 16LD_ellip_ and 16LD_ellip_HS_ ThermalMR RF applicators afforded a maximum SAR_max(TV)_ *=* 41 W/kg. This was slightly superior to that of the 16LD_cir_ and 16LD_cir_HS_ variants, which showed maximum SAR_max(TV)_ = 40 W/kg. Notwithstanding this minor difference, all 16LD ThermalMR RF applicators outperformed the 8D and the 8L variants except ellip_HS, which achieved SAR_max(TV)_ = 41 W/kg for the 16LD and 8D horse-shoe configurations. Values are listed in Table 3.

Results of the SAR amplification factor (SAF) are depicted in a color-coded heatmap for all the ThermalMR RF applicators (Figure 10B, left), and spider plots comparing the three designs (16LD, 8D, 8L) across all four configurations (Figure 10B, right). The 16LD_ellip_HS_ variant showed the highest SAF (4.78), followed by 16LD_cir_HS_ (SAF = 4.69); the 16LD_cir_ and 16LD_ellip_ variants had SAF = 3.95 and SAF = 4.22 (Figure 10B, left). The 8D RF applicators also showed superior SAF values for the horse-shoe configurations, compared to the 360° variants. This observation does not come as a surprise; unlike the 360° variants most of the healthy tissue, e.g., eyes, nose, chin, is not exposed to maximum SAR for the horse-shoe arrays. The overall SAF gain of the 8D variants (8D_cir_ = 5.39, 8D_cir_HS_ = 6.25, 8D_ellip_ = 5.36, 8D_ellip_HS_ = 6.02) is plausible because of the overall reduced mean, max and min achieved SAR_10g_ compared with the 16LD variants (Figure 10B, left). All 8L RF applicators showed SAF~2, indicating that there was no greater power deposition in the TV compared with the healthy tissue. By contrast, the 16LD and 8D arrays achieved higher SAF, indicating better SAR deposition in the TV with preservation of remote healthy tissue from SAR exposure.

Quantification of the hotspots-to-target quotient (HTQ) revealed that all the 16LD and 8D RF applicators showed an HTQ of ~0.88 (Figure 10C, left). This indicates that the maximum SAR_10g_ in the hotspot of the healthy tissue does not exceed the mean SAR_10g_ of the TV. The 16LD_cir_ and 16LD_cir_hs_ RF applicators showed HTQ = 0.83, 0.84 and HTQ = 0.8, 0.79 for the 16LD_ellip_ and 16LD_ellip_hs_ variants. The 8D counterparts showed HTQ = 0.74, 0.72 for the horse-shoe variants 8D_cir_HS_ and 8D_ellip_HS_ and HTQ = 0.84, 0.88 for 8D_cir_, 8D_ellip_. All 8L variants showed an HTQ > 1: 8L_cir_ = 2.11, 8L_cir_HS_ = 2.42, 8L_ellip_ = 2.15, 8L_ellip_HS_ = 2.09. The ellip_HS configuration showed the best results for the 16LD, 8D and 8L variants (Figure 10C, left). 

**Figure 11:**
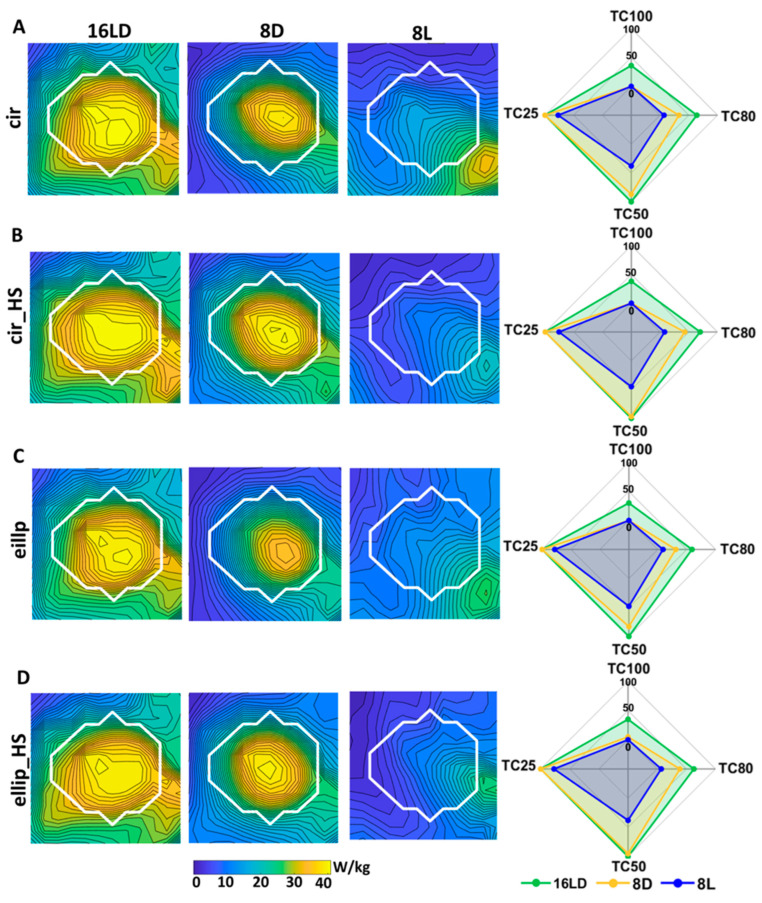
Magnified field of view (FoV) of RF power deposition (SAR_10g_) obtained from targeted RF heating optimization in the tumor target volume. The peak SAR_10g_ within the tumor TV (white line) is shown for 16LD, 8D, 8L ThermalMR RF applicators for the (**A**) cir, (**B**) cir_HS, (**C**) ellip, (**D**) ellip_HS variants. Quantification of tumor coverage (TC) is shown in the spider plots on the right, which show the fraction of the tumor enclosed within the 25, 50, 80 and 100% isolines of peak SAR_10g_ (TC25, TC50, TC80, TC100, respectively) for each ThermalMR RF applicator. The 16LD ThermalMR RF applicator achieved much greater TC50, TC80 and TC100 than the 8D. The 8L ThermalMR RF applicators failed to achieve TC25 = 100%, and in general had much lower TC values compared to 16LD and 8D ThermalMR RF applicators.

A magnified view of the tumor TV is shown in Figure 11A–D, to better highlight differences in tumor coverage (TCx) of RF power deposition among all ThermalMR RF applicators, along with spider plots showing the TCx quantification in the last column. All 16LD and 8D variants showed T25 = 100%, i.e., 100% of the tumor TV received 25% of peak SAR_10g_. The 8L variants showed less TC25: 8L_cir_ = 77%, 8L_cir_HS_ = 76%, 8L_ellip_ = 78%, 8L_ellip_HS_ = 77%. The 16LD RF applicators outperformed the 8D variants in TC50, TC80 and TC100. All 16LD RF applicators achieved T50 = 100%; the 8D_cir_, 8D_cir_HS_, 8D_ellip_ and 8D_ellip_HS_ achieved T50 = 87, 97, 83, 95%, respectively; the 8L_cir_, 8L_cir_HS,_ 8L_ellip_ and 8L_ellip_HS_ achieved T50 = 38, 45, 48, 38%, respectively. 16LD_cir_, 16LD_cir_HS_, 16LD_ellip_ and 16LD_ellip_HS_ achieved TC80 = 64, 70, 59, 63%, respectively, which is an 93, 63, 94 and 62% TC80 improvement over the 8D counterparts of TC80 = 33, 43, 31, 39% for 8D_cir_, 8D_cir_HS_, 8D_ellip_ and 8D_ellip_HS_ , respectively. The 8L_cir_, 8L_cir_HS_, 8L_ellip_ and 8L_ellip_HS_ achieved much less TC80 = 7, 8, 9, 7%, respectively. 16LD_cir_, 16LD_cir_HS_, 16LD_ellip_ and 16LD_ellip_HS_ showed an 93%, 63%, 94% and 62% TC80 improvement over the 8D counterparts. No 8D or 8L RF applicators achieved any TC100, and 8L showed overall much lower values. But 16LD_cir_, 16LD_cir_HS_, 16LD_ellip_ and 16LD_ellip_HS_ yielded TC100 of 36%, 38%, 30% and 35%, respectively. Among the 16LD RF applicators, the horse-shoe configurations achieved better T80 and TC100 than the 360° variants.

**Figure 12:**
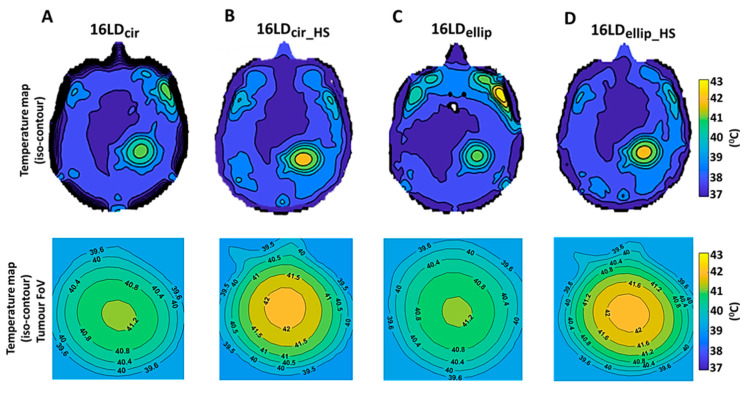
Iso-contour temperature distributions maps of Duke’s head (central axial slice) based on SAR_10g_ deposition from targeted RF heating. Top row of iso-contour temperature maps of 16LD ThermalMR RF applicators (**A**) 16LD_cir_, (**B**) 16LD_cir_HS_, (**C**) 16LD_ellip_, (**D**) 16LD_ellip_HS_, showing the temperature increase from 37.4 °C basal body temperature. The 16LD_cir_ (**A**) and 16LD_ellip_ (**C**) ThermalMR RF applicators show unwanted hotspots of ~42 °C and ~43 °C in the right facial muscles. Correspondingly, magnified field of view (FoV) (bottom row) of highlighted tumor region showing the detail temperature coverage results of T_40°C_, T_41°C_, T_42°C_. Magnified FoV of the tumor region shows that the horse-shoe ThermalMR RF applicators yielded a higher temperature increase (**B**) 16LD_cir_HS_: ΔT = 4.9 °C, (**D**) 16LD_ellip_HS_: ΔT = 4.8 °C from 37.4 °C basal body temperature than the (**A**) 16LD_cir_ and (**C**) 16LD_ellip_ ThermalMR RF applicators, both of which failed to achieve any target volume with T_42°C_ in contrast to the horse-shoe ThermalMR RF applicators (T_42°C_~20%).

This is also illustrated in the iso-contour temperature maps comparing the four 16LD ThermalMR RF applicators (Figure 12A–D, top), with a magnified view of the temperature achieved within the tumor TV (Figure 12A–D, bottom). The horse-shoe variants 16LD_cir_HS_ and 16LD_ellip_HS_ achieved T_mean_ = 41.5 °C, T_mean_ = 41.3 *°*C, respectively, while the 16LD_cir_ and 16LD_ellip_ variants showed T_mean_ = 40.2 °C and T_mean_ = 40.4 °C inside the tumor. Detailed temperature coverage results show the T_40°C_, T_41°C_, T_42°C_ iso-lines within the tumor region. The horse-shoe variants showed superior performance, with T_42°C_ = ~20%, T_41°C_ = ~70%, T_40°C_ = ~10% in the TV (Figure 12B,D, bottom). The 16LD_cir_ and 16LD_ellip_ showed T_40°C_ = ~75% and T_41°C_ = ~25% but no T_42°C_ (Figure 12A,C, bottom).

**Figure 13:**
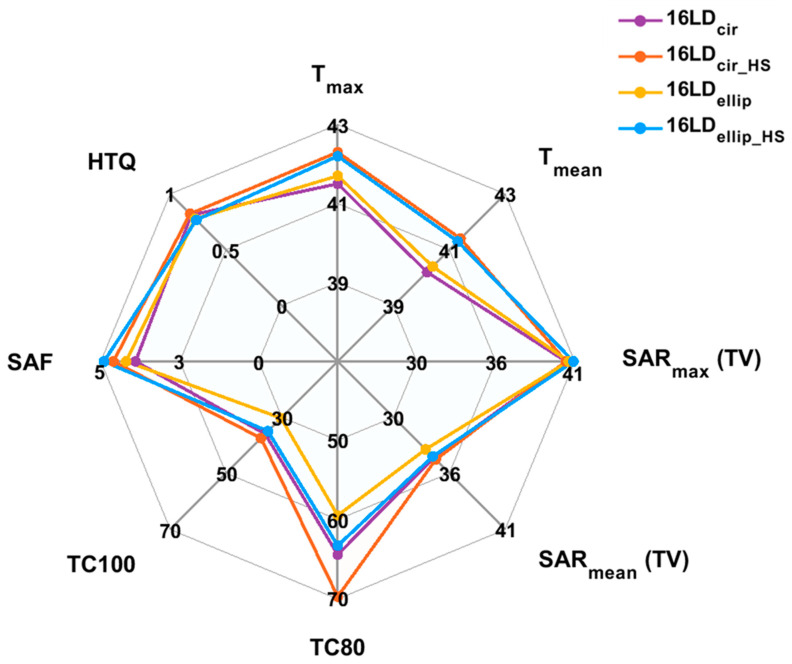
Overall performance evaluation of ThermalMR RF applicators 16LD_cir_, 16LD_ellip_, 16LD_cir_HS_ and 16LD_ellip_HS_ using the metrics maximum and mean SAR (SAR_max(TV)_, SAR_mean(TV)_) and temperature (T_max_, T_mean_) inside tumor, tumor coverage (TC80, TC100), SAR amplification factor (SAF) and hotspot-to-target quotient (HTQ). Both horse-shoe ThermalMR RF applicators 16LD_cir_HS_ and 16LD_ellip_HS_ showed superior TC80 and TC100, higher temperature rise, enhanced SAF and a lower HTQ, compared with the 360° ThermalMR RF applicators 16LD_cir_, 16LD_ellip_.

An overview of all the performance metrics, comparing the 16LD ThermalMR applicators among the four configurations is shown in the spider plots in Figure 13. Both horse-shoe-shaped 16LD variants demonstrated superior tumor coverage versus the 360° counterparts. The greater TC50, TC80 and TC100 performance of the 16LD horse-shoe variants facilitated a higher temperature increase from basal body temperature T = 37.4 °C, 16LD_cir_HS_: ∆T = 4.9 °C, 16LD_ellip_HS_: ∆T = 4.8 °C versus the circular and elliptical 16LD variants.

## 4. Discussion

Our EMF and temperature simulations in realistic human voxel models of brain tumors add to the literature and demonstrate the efficacy of advanced RF applicators that integrate both loop elements and compact SGBT dipole antennas into hybrid building blocks for ThermalMR theranostics. Our results confirm the hypothesis that ThermalMR RF applicators comprising hybrid loop+dipole building blocks outperform dipole-only and loop-only counterparts in both MRI performance and targeted thermal intervention. Unlike our advanced RF applicators equipped with hybrid loop+SGBT building blocks, loop-only antenna arrays cannot deposit adequate RF power to TV located deep inside the head due to their limited penetration depth in lossy tissue. However, loop antennas support deposition of RF power in peripheral regions of the brain or head, benefiting thermal therapy of TVs located close to the surface. These simulation results provide a crucial technical foundation for the implementation and application of 16 channel LD RF applicators and a springboard for ThermalMR-based theranostics of brain tumors. 

Of the 16LD RF applicators, the horse-shoe configurations showed superior tumor coverage and temperature rise inside the tumor compared with the circular 360°-coverage configurations inside the tumor. The horse-shoe configurations have the additional advantages of improved patient comfort by eliminating loop+SGBT building blocks placed close to the eyes, nose and chin, and reduced off-target RF power deposition in the eyes and facial orbit, which benefits RF safety. The antenna arrangement of the horse-shoe variants resulted in a higher SAF and lower HTQ compared with the 360° configurations. Our temperature simulations revealed hotspots in less well-perfused head regions such as the right-side facial muscle tissue, with ~42 °C (16LD_cir_) and ~43 °C (16LD_ellip_). Nevertheless, these hotspots showed temperatures below the standard upper limit for muscle, fat, and bone tissues (T_lim_ < 44 °C) [92], which saves healthy tissue from high-temperature exposure. These findings confirm our second hypothesis, that the horse-shoe shaped ThermalMR RF applicators provide better support for MRI and targeted RF heating performance than the 360°-coverage configurations. These advantages suit the clinical needs of ThermalMR theranostics of brain tumors. 

We applied a time and frequency multiplexed vector field shaping (MVFS) algorithm to optimize the constructive interference of the electric fields used for targeted RF heating [84]. This algorithm provides multiple excitation modes for appropriate frequencies that do not need to be a priori defined or selected, but rather can adapt to the specific tumor TV for each patient. This is useful for RF applicators, like the self-grounded bow-tie and multi-resonant loop used in our study that are capable of delivering RF power over multiple or broadband frequencies. Although the theoretical maximum number of excitation modes is equal to the total number of antenna elements in the RF applicator, the actual number of excitation modes used in real-world applications of ThermalMR is lower than the maximum. Depending on the ThermalMR RF applicator variants, the algorithm identified specific time and frequency-multiplexed individual modes at different distinct frequencies (250–450 MHz) as the optimum solution to create the overall resultant SAR pattern in the target region. The strength of the MVFS optimization approach is in the utilization of excitations over different frequencies to deposit the maximum power uniformly in the tumor target volume at the desired location. Furthermore, the excitations can be adapted to the size and geometry of tumor TVs to suit the clinical needs of individual patients, and support a ‘personalized medicine’ approach without the need for patient-specific RF applicator hardware.

In this study, we used a single-ring array of LD building blocks, which might constrain MRI to limited anatomical coverage of the head. This potential constraint could be addressed by adding a second ring of LD building blocks along the cranial-caudal axis, which would improve the head coverage of the ThermalMR RF applicator. However, this is not necessarily required since the single-ring RF applicator can be moved along the cranial-caudal axis so that the tumor position is located in the center of the ring, providing a viable and cost-effective approach adapted to the needs of the patient. Incorporating novel metamaterial-surfaces or high dielectric pads offers another alternative approach and research direction to improve MRI performance and heating efficacy [97,98,99,100,101]. Metamaterial planar surfaces (metasurface) are conceptually appealing for pursuing the development of RF applicators for ThermalMR-based theranostics of brain tumors due to the extra degrees of freedom for shaping electromagnetic fields they can provide [97,98,99,100,101]. This benefit could be exploited for further SAR_10g_ reduction and B_1_^+^ uniformity improvements for MRI and targeted RF power deposition in deep-seated brain tumors [101,102].

Our thermal intervention results reveal the relationship between temperature change, SAR and the effective perfusion. If the tumor TV includes large blood vessels or anatomical regions with high perfusion, more RF power deposition would be required to achieve the desired temperature increase. To increase the temperature in the brain beyond the (T_max_ = ~42.3 °C) achieved in the current study would require SAR > 40 W/kg. Z. Rijnen et al. reported the use of RF power deposition up to 96–178 W/kg inside the tumor region in head and neck cancer treatment [3,22]. However, additional SAR deposition might create more unwanted hotspots. Implementation of a water bolus with cooling circulation placed between the head and the RF array to cool down the less well-perfused facial muscle regions could help limit hotspot generation. Several studies have suggested the use of a water bolus to improve the quality of thermal therapy [71,77,78,92,103,104]. Another way to reduce SAR in unwanted hotspots is to incorporate novel metamaterial absorbers between the RF applicator and head to absorb SAR from hotspots in healthy tissue [102]. Our advanced hybrid loop-dipole ThermalMR RF applicators are also compatible with these approaches.

The physical processes of E-field interference and heat distribution inside the body and brain are complex and heterogeneous. This limits the accuracy with which temperature distributions in heterogeneous perfused tissue can be predicted in thermal models. Nevertheless, numerous applications can benefit from thermal modeling [29,103]. The limitations of existing RF hyperthermia systems can be overcome by developing new simulation tools for optimizing clinical treatments, considering temperature-dependent effects on blood perfusion, by using noninvasive temperature measurement with MR-thermometry. This measurement is also facilitated by an appropriately designed ThermalMR RF applicator. Developing a realistic tumor model remains a challenge for numerical simulations, due to the complex pathophysiology of tumors. The structure of tumor vessels exhibits irregular branching and twisting where blood flow does not follow a constant, unidirectional path and not all vessels are perfused continuously [73]. These complexities limit the accuracy of blood perfusion coefficients used in the temperature simulations of the tumor model. To advance thermal simulations of tumor models, patient-specific vasculature networks can be derived from MR angiography and incorporated into the model [3,85,103]. Also, patient-specific perfusion maps can be derived from perfusion-weighted MRI. Tumors can also exhibit disrupted cellular and extracellular composition with altered dielectric properties that can be heterogeneous even within the tumor itself [74]. MR-based tomography of electric properties can be employed to better model the temperature-dependent conductivity and permittivity of tumors in the simulations [105]. Notwithstanding these potential future advances, numerical EMF and temperature simulations have become essential for assessing the performance of ThermalMR RF applicators and for temperature dose management. The strength of our numerical thermal modeling is that prior to a treatment session, a selection of patient-specific ThermalMR RF applicator settings can be determined, together with specific RF power optimization planning and thermal dose management. This can be used to maximize temperature in the target volume using SAR as a primary input for the planning, evaluation, and optimization of ThermalMR-based theranostics of brain tumors [71,85,94].

## 5. Conclusions

This work demonstrates the feasibility and applicability of 16-channel hybrid ThermalMR RF applicators combining compact SGBT dipole and loop antennas for ThermalMR theranostics of deep-seated brain tumors at ultrahigh magnetic field strengths of 7.0 T, 9.4 T and 10.5 T. Targeted RF heating of brain tumors can be adapted in a patient-specific manner without changing the RF applicator hardware, by utilizing the multi-resonant and broadband characteristics of the loop+SGBT dipole building blocks in conjunction with the time and frequency multiplexed vector field shaping algorithm. The enhanced RF power deposition and temperature rise up to ~42.3 °C inside the tumor target volume meet the temperature criteria for adjunct hyperthermia therapy of glioblastoma multiforme. Our EMF and temperature simulations establish a solid technical foundation for the development and construction of hybrid loop+SGBT dipole ThermalMR RF applicators. Such numerical simulations are a mandatory precursor for future in vivo studies and provide a rigorous framework en route to clinical ThermalMR-based theranostics of brain tumors. This also has implications for investigating fundamental questions in biology, molecular medicine and molecular imaging. ThermalMR can potentially be used to investigate (patho)physiological processes, opening an entirely new research field of thermal phenotyping of cancer. “Can stages of tumor progression and therapy response be characterized by thermal profiles?”; “What are the links between MRI biomarkers, thermal profiles and the molecular signatures of brain tumors?” ThermalMR has the potential to offer an answer to such questions.

## Figures and Tables

**Table 1:** Input power for temperature simulation from individual excitation mode (M) power obtained from targeted RF heating optimization for 16LD thermalMR RF applicators.

ThermalMR RF Applicator	Individual Excitation Mode (M) Power				
16LD_cir_	300 MHz	350 MHz	450 MHz	450 MHz	Total
	0.65 W	31.48 W	24.01 W	15.12 W	71.26 W
16LD_cir_HS_		350 MHz	450 MHz	450 MHz	Total
		21.33 W	34.21 W	15.23 W	70.77 W
16LD_ellip_	300 MHz	350 MHz	450 MHz	450 MHz	Total
	14.55 W	15.84 W	22.72 W	18.21 W	71.32 W
16LD_ellip_HS_		350 MHz	450 MHz	450 MHz	Total
		18.89 W	32.66 W	18.75 W	70.3 W

**Table 2:** Summary of statistical analysis of B_1_^+^ transmission field for MRI performance of the ThermalMR RF applicators.

ThermalMR RF Applicator		Mean 7 T	Max 7 T	Min 7 T	* *p*-Value (vs. 16LD) 7 T	Mean 9.4 T	Max 9.4 T	Min 9.4 T	* *p*-Value (vs. 16LD) 9.4 T	Mean 10.5 T	Max 10.5 T	Min 10.5 T	* *p*-Value (vs. 16LD) 10.5 T
cir	16LD	19.42	21.39	16.14		20.87	24.74	15.26		21.25	26.18	13.90	
	8D	12.89	14.63	10.27	1.05 × 10^−83^	16.18	19.07	10.83	8.42 × 10^−70^	17.26	21.30	10.23	1.56 × 10^−43^
	8L	14.63	16.26	12.28	1.09 × 10^−83^	13.4	16.10	9.72	3.33 × 10^−83^	12.69	15.86	8.79	5.70 × 10^−82^
cir_HS	16LD	18.33	20.22	14.69		20.01	24.06	14.02		20.33	24.70	12.94	
	8D	14.36	16.03	10.80	3.23 × 10^−80^	16.82	20.14	11.08	3.61 × 10^−46^	16.84	20.96	10.16	9.48 × 10^−34^
	8L	11.57	12.69	9.90	1.03 × 10^−83^	11.86	14.06	8.63	1.07 × 10^−83^	11.51	14.11	7.78	3.80 × 10^−83^
ellip	16LD	19.77	21.95	16.66		20.03	23.82	14.80		20.40	25.20	13.53	
	8D	12.95	14.54	10.71	1.05 × 10^−83^	15.99	18.52	11.30	9.25 × 10^−65^	17.04	20.64	10.66	1.71 × 10^−36^
	8L	15.07	16.78	12.17	1.31 × 10^−83^	12.41	15.22	8.34	2.04 × 10^−83^	11.70	15.03	7.16	2.34 × 10^−82^
ellip_HS	16LD	18.37	20.47	14.48		19.74	23.44	13.85		20.66	25.54	13.47	
	8D	14.11	15.52	10.93	8.13 × 10^−82^	16.43	19.18	10.99	1.31 × 10^−49^	17.29	21.20	10.61	3.08 × 10^−34^
	8L	11.89	13.83	8.72	1.05 × 10^−83^	11.51	13.99	7.44	1.11 × 10^−83^	11.77	14.92	7.21	4.94 × 10^−82^

* *p*-values are from comparison to 16LD.

**Table 3:** Summary of statistical analysis of the targeted RF heating performance of the ThermalMR RF applicators using the metric total SAR_10g_ inside the tumor volume.

ThermalMR RF Applicator	Mean		Max	Min	* *p*-Value (vs. 16LD)
cir	16LD	34.1	40.4	22.9	
	8D	27.1	38.8	12.4	5.32 × 10^−5^
	8L	10.8	24.2	1.33	3.96 × 10^−8^
cir_HS	16LD	34.7	40.4	20.3	
	8D	30.9	40.3	17.3	6.64 × 10^−5^
	8L	5.57	12.9	0.712	1.18 × 10^−7^
ellip	16LD	33.6	40.6	20.5	
	8D	25.0	35.4	10.5	5.85 × 10^−5^
	8L	9.14	19.3	1.47	3.52 × 10^−7^
ellip_HS	16LD	34.4	40.9	20.3	
	8D	30.1	40.6	17.2	1.9 × 10^−3^
	8L	7.69	18.1	1.15	1.56 × 10^−7^

* *p*-values are from comparison to 16LD.

## Data Availability

All data can be found in the text.

## Supplementary Materials

The following supporting information can be downloaded at: https://www.mdpi.com/article/10.3390/cancers15082303/s1, Table S1: The dielectric and thermal properties of the tumor and healthy tissues used for the human voxel model Duke.

## Abbreviations

MR	magnetic resonance
MRI	magnetic resonance imaging
ThermalMR	thermal magnetic resonance
MRTh	MR thermometry
RF	radiofrequency
UHF	ultrahigh field
HT	hyperthermia
RT	radiotherapy
WHO	World Health Organization
GBM	glioblastoma multiforme
CNS	central nervous system
TMTP	thermalMR treatment planning
E-field	electric Field
B_1_^+^	RF transmission field used for MRI
B₀	main static magnetic field of MRI scanner
SAR	specific absorption rate
SAR_10g_	10 g averaged SAR distribution
EMF	electromagnetic field
SGBT	self-grounded bow-tie
LD	loop+SGBT dipole building block
L	loop building block
D	SGBT dipole building block
16LD	hybrid 16-channel loop+SGBT dipole ThermalMR RF applicator
8D	8-channel SGBT dipole ThermalMR RF applicator
8L	8-channel loop ThermalMR RF applicator
cir	circular array
cir_HS	circular horse-shoe array
ellip	elliptical array
ellip_HS	elliptical horse-shoe array
16LD_cir_	circular array variant of 16LD
16LD_cir_HS_	circular horse-shoe array variant of 16LD
16LD_ellip_	elliptical array variant of 16LD
16LD_ellip_HS_	elliptical horse-shoe array variant of 16LD
8D_cir_	circular array variant of 8D
8D_cir_HS_	circular horse-shoe array variant of 8D
8D_ellip_	elliptical array variant of 8D
8D_ellip_HS_	elliptical horse-shoe array variant of 8D
8L_cir_	circular array variant of 8L
8L_cir_HS_	circular horse-shoe array variant of 8L
8L_ellip_	elliptical array variant of 8L
8L_ellip_HS_	elliptical horse-shoe array variant of 8L
MVFS	multiplexed vector field shaping
M	excitation mode provided by MVFS algorithm
PLD	power loss density
ROI	region of interest
TV	target volume
TC	tumor coverage
TC25	fraction of the tumor volume enclosed by 25% peak SAR_max_ isolines
TC50	fraction of the tumor volume enclosed by 50% peak SAR_max_ isolines
TC80	fraction of the tumor volume enclosed by 80% peak SAR_max_ isolines
TC100	fraction of the tumor volume enclosed by 100% peak SAR_max_ isolines
HTQ	Hotspot-to-target quotient
SAF	SAR amplification factor
ESHO	European Society for Hyperthermic Oncology
T	temperature
T_mean_	mean temperature inside TV
T_max_	maximum temperature inside TV
T_x°C_	index temperature coverage inside TV
T_40°C_	fraction of the tumor volume enclosed by 40 °C
T_41°C_	fraction of the tumor volume enclosed by 41 °C
T_42°C_	fraction of the tumor volume enclosed by 42 °C
Max	maximum
Min	minimum
CEM	cumulative equivalent minutes
FoV	field-of-view
