# Supplementary material for: Advanced Radio Frequency Applicators for Thermal Magnetic Resonance Theranostics of Brain Tumors
Source: Cancers (Basel). 2023 Apr 14;15(8):2303. doi: 10.3390/cancers15082303 (PMC10137156; doi:10.3390/cancers15082303)
Supplement: Supplementary file 1 [file cancers-15-02303-s001.zip › cancers-2296928-supplementary.pdf]

Table S1: The dielectric and thermal properties of the tumor and healthy tissues used for the human voxel model Duke

Tissue	Relative permittivity	Electrical conductivity	Mass density	Thermal conductivity	Specific heat	Diffusivity	Blood perfusion coefficient	Metabolic rate
		[S/m]	[kg/m^3]	[W/k/m]	[J/k/kg]	[m^2/s]	[W/K/m^3]	[W/m^3]
Air_internal	1			0.02	100			
Artery	64.8198	1.33296	1060	0.51	3824	1.26E-07	1.00E+06	
Blood Vessel Wall	64.8198	1.33296	1060	0.51	3824	1.26E-07	1.00E+06	
Bone (Cortical)	13.2772	0.0869573	1908	0.4	1289	1.56E-07	3400	610
Brain (Grey Matter)	58.5531	0.7156	1045	0.502	3700	1.32E-07	40000	7100
Brain (White Matter)	42.8108	0.429387	1041	0.502	3600	1.35E-07	17280	7100
Cartilage	46.0431	0.56943	1100	0.624	3500	1.73E-07	9000	1600
Cerebellum	57.5983	1.00296	1045	0.502	3700	1.32E-07	40000	7100
Cerebrospinal Fluid	71.7086	2.23818	1007	0.6	4187	1.43E-07		
Commissura Anterior	42.8108	0.429387	1041	0.502	3600	1.35E-07	17280	7100
Commissura Posterior	42.8108	0.429387	1041	0.502	3600	1.35E-07	17280	7100
Connective Tissue	47.5897	0.54809	1027	0.201	2500	8.84E-07	1700	300
ear_cartilage	46.0431	0.56943	1030	0.624	3500	1.73E-07	9000	1600
ear_skin	48.0975	0.665799	1100	0.293	3500	7.61E-08	9100	1620
Esophagus	68.0198	0.987485	1040	0.53	3500	1.46E-07	9000	1600
Eye (Cornea)	60.1932	1.17225	1076	0.52	3793	1.27E-07	2585	
Eye (Lens)	37.8697	0.362949	1090	0.4	3664	1.00E-07		
Eye (Sclera)	58.2086	0.989943	1032	0.4	3000	1.29E-07	1961	
Eye (Vitreous Humor)	69.0084	1.52309	1009	0.624	3932	1.57E-07		
Fat	11.6766	0.0785219	911	0.201	2500	8.84E-08	1700	300
Hippocampus	58.5531	0.7156	1045	0.502	3700	1.32E-07	40000	7100
Hypophysis	61.943	0.864093	1066	0.624	3761	1.58E-07	360000	64000
Hypothalamus	58.5531	0.7156	1050	0.624	3761	1.58E-07	360000	64000
Intervertebral Disc	46.7393	0.926542	1100	0.624	3500	1.73E-07	9000	1600
Larynx	46.0431	0.56943	1100	0.624	3500	1.73E-07	9000	1600

Mandible	13.2772	0.0869573	1990	0.4	1289	1.56E-07	3400	610
Medulla Oblongata	57.5983	1.00296	1046	1.13	3675	2.99E-07	40000	7100
Bone Marrow (Red)	11.9831	0.178457	1028	0.22	2700	7.93E-08	32000	5700
Midbrain	57.5983	1.00296	1046	1.13	3675	2.99E-07	40000	7100
Mucous Membrane	57.5958	0.783464	1050	0.34	3150	1.03E-07	9000	1600
Muscle	57.5958	0.783464	1041	0.53	3546	1.44E-07	2700	480
Nerve	36.0647	0.43294	1038	0.46	3664	1.21E-07	40000	7100
Pharynx	1		1	0.02	1000			
Pineal Body	61.943	0.864093	1053	0.624	3761	1.58E-07	360000	64000
Pons	57.5983	1.00296	1046	1.13	3675	2.99E-07	40000	7100
SAT	11.6766	0.0785219	910	0.201	2500	8.84E-08	1700	300
Skin	48.0975	0.665799	1109	0.293	3500	7.61E-08	9100	1620
Skull Cortical	13.2772	0.0869573	1990	0.4	1289	1.56E-07	3400	610
Spinal Cord	36.0647	0.43294	1075	0.46	3664	1.21E-07	40000	7100
Tendon\Ligament	47.5897	0.54809	1142	0.5	3500	1.29E-07	3750	480
Tooth	13.2772	0.0869573	2180	0.4	1289	1.56E-07	3400	610
Thalamus	58.5531	0.7156	1045	0.502	3700	1.32E-07	40000	7100
Tongue	58.2068	0.759415	1090	0.53	3546	1.44E-07	2700	480
Trachea	44.6848	0.6237	1080	0.47	3664	1.17E-07	9000	1600
Trachea Lumen	1		1	0.02	1000			
vein	64.8198	1.33296	1060	0.51	3824	1.26E-07	1.00E+06	
Vertebrae	13.2772	0.0869573	1990	0.4	1289	1.56E-07	3400	610
Tumor	66.5	1.15	1025.5	0.5	3600	1.35E-07	12096	3000
